# Clinical and prognostic significance of e1a2 BCR-ABL1 transcript subtype in chronic myeloid leukemia

**DOI:** 10.1038/bcj.2017.62

**Published:** 2017-07-14

**Authors:** Z Gong, L J Medeiros, J E Cortes, L Zheng, J D Khoury, W Wang, G Tang, S Loghavi, R Luthra, W Yang, H M Kantarjian, S Hu

**Affiliations:** 1 Departments of Hematopathology, The University of Texas MD Anderson Cancer Center, Houston, TX, USA; 2 Departments of Leukemia, The University of Texas MD Anderson Cancer Center, Houston, TX, USA; 3 Department of Hematology, Shengjing Hospital of China Medical University, Shenyang, China

Chronic myeloid leukemia (CML) is characterized by the presence of *BCR-ABL1* fusion gene. In over 95% of CML patients, the typical *BCR-ABL1* transcript subtypes are e13a2 (b2a2), e14a2 (b3a2) or expression of both simultaneously. Other less frequent transcript subtypes, such as e1a2, e2a2, e6a2, e19a2, e1a3, e13a3 and e14a3, have been sporadically reported.^1^ Different subtypes of *BCR-ABL1* transcripts encode fusion proteins with different sizes that may lead to different disease phenotypes. The e13a2 and e14a2 transcripts encode P210 BCR-ABL1 proteins with slightly different sizes. Patients with the e14a2 transcript have a significantly higher platelet count than those with the e13a2 transcript.^2, 3, 4^ Patients with the e19a2 transcript, which encodes P230, often present with prominent neutrophilic maturation or thrombocytosis, whereas patients with the e1a2 transcript, which encodes P190, often present with monocytosis, absence of basophilia and a tendency to progress to lymphoid blast phase (BP).^5, 6, 7^

Despite the established link between *BCR-ABL1* transcript subtypes and disease phenotype, the impact of different transcript subtypes on the disease course and patient outcome is less clear. Several small case series have suggested an association of e1a2 transcript subtype with a less favorable response and a faster disease progression. However, the conclusions of these studies are limited by the low number of patients.^5, 8, 9^ To investigate the significance of the e1a2 transcript subtype, we examined a cohort of 2322 CML patients treated with tyrosine kinase inhibitors (TKIs), and analyzed the frequency, risk of blastic transformation, treatment response and outcomes of patients with this transcript subtype.

CML patients that met following selection criteria were included in this study: (1) receiving TKIs as part of frontline therapy; (2) age at diagnosis ⩾18 years; and (3) subtype of *BCR-ABL1* transcripts confirmed by reverse transcription PCR. BP was defined by the presence of ⩾20% blasts in peripheral blood or bone marrow.

The study group included 1326 men and 996 women with a median age of 48 years (range, 18–88) at diagnosis. The median follow-up time was 109.8 months (range, 0–221.6 months). A total of 2266 (97.6%) patients had the typical transcripts, including e13a2 (*n*=920), e14a2 (*n*=950) and both e13a2 and e14a2 (*n*=396). Forty-one (1.8%) patients had the e1a2 transcript. Other rare transcript subtypes included e14a3 (*n*=4), e13a3 (*n*=2), e6a2 (*n*=1) and others (*n*=8). The clinical characteristics of patients with the e1a2 vs typical transcripts are compared in Table 1a. The median age was 59 years at diagnosis of CML in patients with the e1a2 transcript vs 48 years in patients with the typical transcripts (*P*<0.001).

Of CML patients with the e1a2 transcript, 16 had BP, 2 had accelerated phase by blast count and 23 had chronic phase at initial diagnosis. Of the 25 patients who did not have BP initially, 10 had a peripheral blood count and differential count prior to treatment available. All 10 patients had relative and absolute monocytosis at initial presentation, with a median percentage of 11.5% (range, 5–36%), and 7 patients had monocytosis over 10%. Of a total of 8 patients (8/41) with the e1a2 transcript who developed myeloid BP, expression of monocytic markers in the blast population was observed in three patients.

Patients with the e1a2 transcript had a higher frequency of additional chromosomal abnormalities (ACAs) than those with the typical transcripts: 46.3% (19/41) vs 25.2% (572/2266), *P*=0.002. Of these with known emerging time of ACAs, 9/37 (24.3%) vs 177/2092 (8.5%) patients with the e1a2 and typical transcripts had ACAs at diagnosis, respectively (*P*<0.001), and 6/37 (16.2%) vs 221/2092 (10.6%) acquired ACAs during therapy respectively (*P*=0.27). There was no significant difference in the frequency of high-risk ACAs, including 3q26.2 rearrangement, −7/7q- and i(17q) (*P*=0.33), or complex ACAs between those with the e1a2 and those with the typical transcripts (*P*=0.13).

CML patients with the e1a2 transcript had a significantly worse overall survival (OS) than patients with the typical transcripts with a median OS of 69.5 vs 206.8 months, respectively (Figure 1a1, *P*<0.001). After excluding patients who had BP initially or ACAs initially and those with unknown emerging time of ACAs, the e1a2 transcript subtype remained an adverse prognostic factor (Figure 1a2).

Patients with the e1a2 transcript were more likely to develop BP than patients with the typical transcripts: 25/41 (61.0%) vs 399/2266 (17.6%), respectively (*P*<0.001). When stratified by the timing of BP, 16/41 (39.0%) vs 78/2266 (3.4%) patients with the e1a2 vs typical transcripts presented in BP at initial diagnosis, respectively (*P*<0.001). In patients who did not present in BP initially, 9/25 (36.0%) vs 321/2188 (14.7%) patients with the e1a2 vs typical transcripts developed BP, respectively, after a median follow-up of 90.3 months (*P*<0.001). The hazard ratio for the e1a2 transcript to increase the risk of progression to BP was 2.45 (95% confidence interval: 1.44–4.18). Furthermore, the lineage of BP in patients with the e1a2 transcript was more likely to be lymphoid or mixed phenotype: 17/25 (68.0%) vs 124/399 (31.1%) in those with the typical transcripts (*P*<0.001).

In patients who did not present in BP initially, those with the e1a2 transcript had a shorter latency from initial diagnosis to blastic transformation (Figure 1b1). The cumulative probability of blastic transformation at 18 months was 29.2% vs 6.4% in patients with the e1a2 vs typical transcripts, respectively (*P*<0.001). After excluding patients with ACAs initially and those with unknown emerging time of ACAs, the e1a2 transcript remained associated with a faster progression to BP (cumulative probability of BP at 18 months: 21.1% vs 6.1%, *P*=0.018, Figure 1b2). The significant difference in the latency of blastic transformation in these two groups translated into a significant difference in transformation-free survival (TFS) with a median 107.8 vs 202.9 months (*P*=0.006), respectively, when patients with ACAs initially and those with unknown emerging time of ACAs were included (Figure 1c1), and 107.8 vs 206.8 months (*P*=0.033), respectively, when patients with ACAs initially and those with unknown emerging time of ACAs were excluded (Figure 1c2).

In multivariate analyses with covariates including sex, age group, disease stage at diagnosis, ACAs prior to blastic transformation and transcript subtypes, the e1a2 transcript subtype independently predicted both a poorer OS (hazard ratio 2.09, *P*=0.002) and TFS (hazard ratio 2.05, *P*=0.014) (Table 1b).

We then investigate the impact of the e1a2 transcript on treatment responses. Patients with the e1a2 transcript were both significantly slower and less likely to achieve complete cytogenetic remission (CCyR) than those with the typical transcripts, with a median time to CCyR of 53.1 vs 18.8 months, respectively (Figure 1d1, *P*=0.003). The overall CCyR rate was 33.3% (8/24) vs 66.5% (1430/2150) in patients with the e1a2 vs typical transcripts, respectively, after a median follow-up time of 53.0 months. When patients with BP initially or ACAs initially and those with unknown emerging time of ACAs were excluded, the difference in CCyR between two groups remained significant (Figure 1d2, median time to CCyR 17.9 vs 53.1 months, *P*=0.005).

Patients with the e1a2 transcript were both significantly slower and less likely to achieve major molecular remission (MMR) than those with the typical transcripts, with a median time to MMR unreached vs 31.7 months, respectively (Figure 1e1, *P*=0.001). The overall MMR rate was 18.5% (5/24) vs 63.7% (1266/1986) in patients with the e1a2 vs typical transcripts, respectively, after a median follow-up time of 59.5 months. When patients with BP initially or ACAs initially and those with unknown emerging time of ACAs were excluded, the difference in MMR between two groups remained significant (Figure 1e2, *P*=0.001).

Here we investigated the impact of the e1a2 transcript subtype in a cohort of 2322 CML patients treated with TKIs. We found that the e1a2 transcript is rare in CML, ~1.8% in this study. Patients with the e1a2 transcript are diagnosed at an older median age, more likely have monocytosis in chronic phase, and more likely to present in BP initially. Those who do not present in BP initially have a higher risk of subsequent progression to BP, an inferior cytogenetic and molecular response to TKI therapy, and an adverse OS and TFS. In multivariate analysis, the e1a2 transcript subtype independently predicts inferior OS and TFS.

A rapidly growing list of parameters is included as criteria for defining accelerated phase of CML in the 2016 revision to the World Health Organization classification of myeloid neoplasms. These parameters include clinical findings (counts of white blood cells, basophils, platelets and blasts, and splenomegaly), cytogenetic data (such as major-route ACAs at diagnosis and any ACAs acquired during therapy) and response criteria (such as ABL1 mutation).^10^ However, it has been shown that the impact of most of the clinical parameters listed above has been minimized in TKI era.^11^ Similarly, although implicated in disease progression and poor outcome of CML patients, not all major-route ACAs or ABL1 mutations detected at diagnosis or acquired during therapy are equally prognostically significant in TKI era.^12, 13, 14, 15^ The data in this study indicate that the e1a2 transcript subtype emerges as a new high-risk factor for disease progression. It may be helpful to include e1a2 transcript subtype among the ever-changing list of criteria for accelerated phase in a future classification scheme of CML.

## Figures and Tables

**Figure 1:**
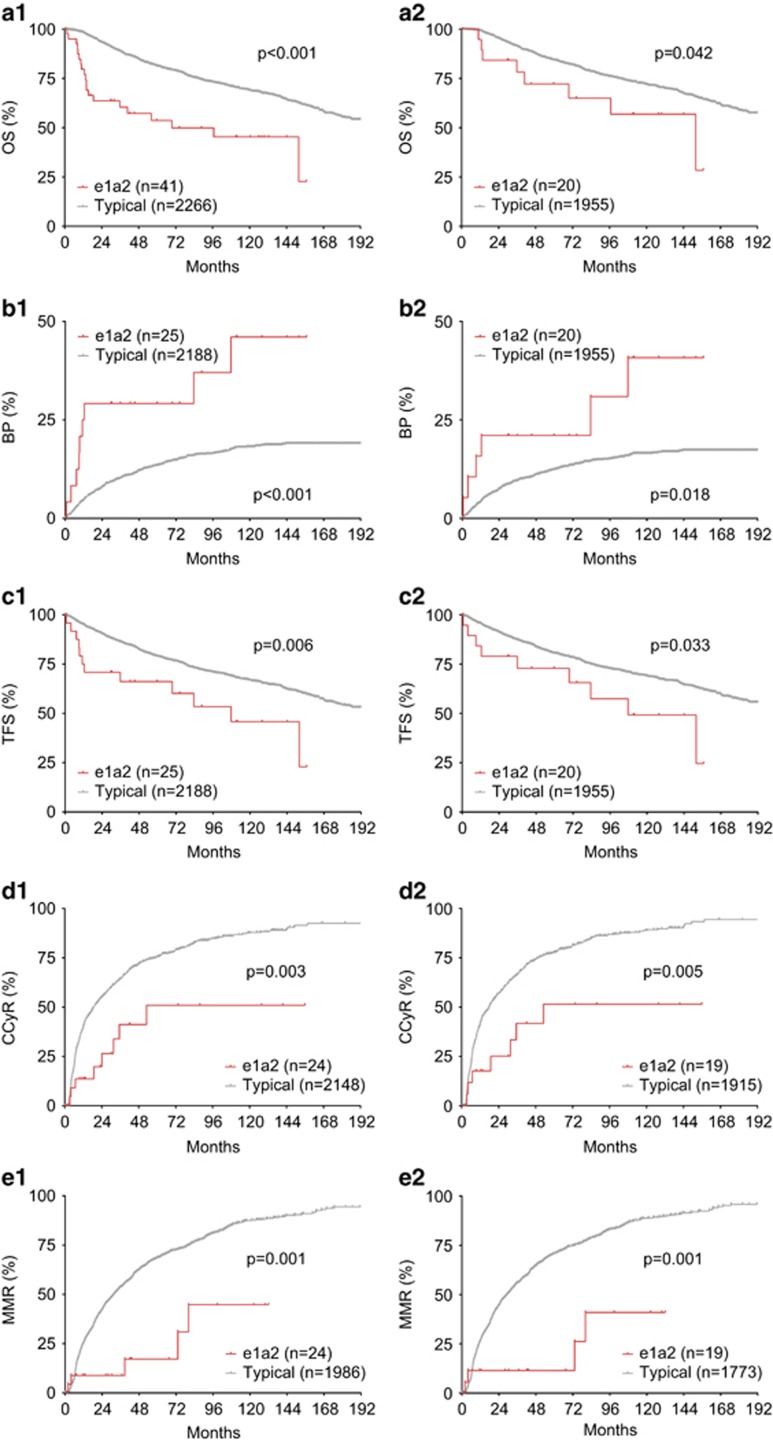
Impact of e1a2 transcript subtype in CML. (**a**1) OS of CML patients with e1a2 vs typical transcripts in the entire cohort. (**a**2) OS of CML patients with e1a2 vs typical transcripts after excluding those with BP initially or ACAs initially and those with unknown emerging time of ACAs. (**b**1, **b**2) Time course of blastic transformation in CML patients with e1a2 vs typical transcripts before (**b**1) and after (**b**2) excluding those with BP initially or ACAs initially and those with unknown emerging time of ACAs. (**c**1, **c**2) TFS in CML patients with e1a2 vs typical transcripts before (**c**1) and after (**c**2) excluding those with BP initially or ACAs initially and those with unknown emerging time of ACAs. (**d**1, **d**2) CCyR in CML patients with e1a2 vs typical transcripts before (**d**1) and after (**d**2) excluding those with BP initially or ACAs initially and those with unknown emerging time of ACAs. A total of 2172 patients had available cytogenetic response data, including 2148 patients with the typical transcripts and 24 patients with the e1a2 transcript. (**e**1, **e**2) MMR in CML patients with e1a2 vs typical transcripts before (**e**1) and after (**e**2) excluding those with BP initially or ACAs initially and those with unknown emerging time of ACAs. A total of 2010 patients had available molecular response data, including 1986 with the typical transcripts and 24 with the e1a2 transcript.

**Table 1a:** Characteristics of patients with e1a2 vs typical transcripts

*Characteristics*	*e1a2 transcript (*N=*41)*	*Typical transcripts (*N=*2266)*	P*-value*
*Sex*			
Male	21	1293	0.45
Female	20	973	
			
*Age at Dx*			
Median	59	48	<0.001
Range	18-86	18-88	
			
*Blast immunophenotype*			
Myeloid	8	275	<0.001
Lymphoid	13	114	
Mixed phenotype	4	10	
			
*Stage at Dx*			
BP	16	78	<0.001
Not BP	25	2188	
			
*ACA at Dx*^a^			
No ACA	28	1915	0.003
Single ACA	7	126	
Multiple ACA	2	51	
			
*Best response*^b^			
CCyR and above	8 (33.3%)	1430 (66.5%)	<0.001
MMR and above	5 (18.5%)	1266 (63.7%)	<0.001
			
*Survival (in months)*			
Median OS	69.5	206.8	<0.001
Median TFS^c^	107.8	202.9	0.006
			
*Status at last F/U*			
Dead	20	623	0.003
Alive	21	1643	
			
*Treatment*			
Imatinib	36	1722	0.078
Non imatinib	5	544	
Dasatinib	4	271	
Nilotinib	1	210	
Bosutinib	0	10	
Ponatinib	0	53	
HSCT	7	249	0.219

Abbreviations: ACA, additional cytogenetic abnormality; BP, blast phase; CCyR, complete cytogenetic response; Dx, diagnosis; F/U, follow-up; HSCT, hematopoietic stem cell transplantation; MMR, major molecular response; OS, overall survival; TFS, transformation-free survival.

a In 4 patients with e1a2 transcript and 174 patients with typical transcripts, the time of ACA emergence was unclear.

b Patients who were in BP initially were not included in response evaluation; response achieved after blastic transformation was not included.

c Patients who were in BP initially were not included in calculation of TFS.

**Table 1b:** Multivariate analysis of prognostic parameters in CML

	*OS*			*TFS*		
	*HR*	*95% CI*	P*-value*	*HR*	*95% CI*	P*-value*
Age group	1.703	1.505–1.926	<0.001	1.484	1.315–1.675	<0.001
Male sex	1.311	1.117–1.538	0.001	1.267	1.083–1.483	0.003
Initially in BP	4.191	3.066–5.730	<0.001	NA	NA	NA
ACAs	1.637	1.275–2.101	<0.001	1.807	1.395–2.342	<0.001
e1a2 transcript	2.091	1.319–3.314	0.002	2.046	1.154–3.630	0.014

Abbreviations: ACA, additional cytogenetic abnormality; BP, blast phase; CI, confidence interval; CML, chronic myeloid leukemia; HR, hazard ratio; NA, not applicable; OS, overall survival; TFS, transformation-free survival. Age group: ⩽40, 41–64, ⩾65.
